# Pathogenicity and virulence of Cryptococcus neoformans from an environmental perspective

**DOI:** 10.1080/21505594.2025.2547090

**Published:** 2025-08-14

**Authors:** Arturo Casadevall

**Affiliations:** Department of Molecular Microbiology and Immunology, Johns Hopkins School of Public Health, Baltimore, MD, USA

**Keywords:** Cryptococcus, virulence, pathogenicity, virulence factors

## Abstract

In the past half century *Cryptococcus neoformans* emerged as a major human pathogen leading the World Health Organization to place it among its top four critical priority group. *C. neoformans* is common in the environment resulting in widespread human exposure but disease in immunocompetent hosts is rare. The fungus is endowed with powerful virulence factors that allow it to resist immunological mechanisms for clearance. Despite this, the fungus does not appear to have any need for an animal host in its life cycle and consequently these virulence factors are traits selected for environmental survival that function accidentally as enablers of virulence in susceptible hosts. Considerable progress has been made in understanding its life cycle, ecology, pathogenicity mechanisms, and antigenic composition for vaccine design. In this essay, the problem of cryptococcal pathogenesis and virulence is approached from the viewpoint of considering these processes in the context of its environment and ecology.

## Introduction

In the late 20^th^ century, the fungus *Cryptococcus neoformans* (CN) emerged as a major human pathogen, a development that occurred largely in parallel with the HIV epidemic that resulted in millions of individuals with severe immunosuppression and medical progress that came at the price of immunity producing legions of immunocompromised hosts. By the early 1990s, New York City had over thousand cases of cryptococcosis per year, making CN the most common cause of culture-positive meningitis in that city [1]. In recent years, advances in antiretroviral therapy have reduced the problem of cryptococcosis in patients with advanced HIV infection; however, a new at-risk population has emerged from the increasing number of individuals with impaired immunity due to medical progress in organ transplantation and oncology. Paralleling its rise in medical importance, interest in the biology and pathogenesis of CN has increased dramatically since the 1980s. Today, the cryptococcal field is thriving and second only to those of *Aspergillus* and *Candida* in the number of investigators working on this fungus [2].

In reviewing the literature for this essay, it was apparent that there have been several outstanding reviews in recent years focusing on diverse aspects, such as epidemiology genetics and sexual reproduction [3], antifungal resistance [4], plant interactions [5], sporulation and germination [6], pathogenesis [7], virulence [8,9], capsule structure [10,11], clinical management [12], signaling [13], morphology [14], extracellular vesicles [15], population heterogeneity [16], blood-brain invasion [17], dormancy and latency [18], extrapulmonary dissemination [19], brain invasion [20], vaccines [21], epidemiology and disease outcome [22] and clinical management [23]. Those interested in a review of the literature up to 1998, including the history of cryptococcosis in the 20^th^ century, are referred to a book written by John Perfect and your author [24]. In addition, readers interested in cryptococcosis at the height of the AIDS epidemic are referred to an authoritative review published on the occasion of the centenary of the discovery of CN as a pathogen [25]. Given my desire to do something different from these recent scholarly reviews, this essay attempts to synthesize the available information on pathogenicity and virulence from an environmental perspective. Unfortunately, even with a review of this size, it is not possible to cover all the information that has been generated by the cryptococcal field, and those interested in more specialized topics are referred to the excellent reviews cited above. Furthermore, as will become evident to the reader, an essay of this type requires more breath and synthesis than depth and detail; consequently, some topics have necessarily been treated superficially, but hopefully, sufficient detail is included to alert the reviewer of the importance of the topic, providing a guide to more detailed treatises.

## The microbe

The genus *Cryptococcus* includes several pathogenic fungal species, of which *C. neoformans* is the most important. Historically, the major pathogenic cryptococcal isolates were divided into two varieties: *C. neoformans* var. *neoformans* and *C. neoformans* var. *gattii* but as genomic information accumulated, it became apparent that these varieties were sufficiently different to be reclassified as different species, which in time were then further subdivided into several species [26], with the caveat that they caused similar clinical diseases. Given concerns about the fragmentation and non-continuity of the cryptococcal literature, some cryptococcal investigators, including your author, have argued for referring to these as a species complex rather than adopting further subdivision [27]. At this time, some laboratories (the splitters) embraced the granular subdivision into multiple species, while others (the groupers) embraced the species complex proposal. Hence, for the purposes of this review, references to both *C. neoformans* and *C. gattii* (CG) are referred to, and considered, as species complexes. CN and CG are thought to have originated in Africa [28] and South America [29], respectively. An analysis of the genomic distance between CN and CG suggests that they separated 80–100 million years ago [30], a time that encompasses the breakup of the supercontinent Pangea. The coincidence and overlap of the dates for the separation of the African and South American continents and the emergence of these species has led to the proposal that CN and CG originated from a common ancestor in Pangea, and then, with increasing geographic distance as the continents drifted apart, evolved into different species [30].

CN has a worldwide distribution and is frequent in cities inhabited by pigeon flocks, which provide a preferred niche in the form of excreta, where the fungus can grow to high density. Pigeons were domesticated by humans and transported to all corners of the planet, serving as pets, food, and instruments of communication. Consequently, CN may have had significant human help in colonizing the world. In contrast, CG has a more limited geographic distribution and is found primarily in tropical regions [31]. However, in the late 1990s, an outbreak began in the Vancouver region, indicating that this species had gained a foothold in North America [32]. The arrival of the CG in the Pacific Northwest may have had anthropomorphic help [33]. The Vancouver outbreak strain is closely related to strains in South America, and it was proposed that these could have arrived in the coastal waters of the Pacific Northwest in the ballast tanks of ships passing through the Panama Canal and then deposited on land as a result of the inundation following the 1964 Alaska earthquake and consequent tsunamis [33]. This hypothesis is supported by the finding that *Cryptococcus* spp. manifest considerable buoyancy in seawater, which could facilitate oceanic and coastal water transport [34]. Like its effects on most life on earth, climate change can be anticipated to alter the geographic prevalence of distribution of CN and CG in the global environment [35]. Climate change has been posited as an important contributor to the spread of CG in the pacific northwest [36,37]. GC genotype VG1 has been expanding in the Mediterranean and is predicted to move from coastal regions to internal European locales [38]. Since heat stress is associated with increased rates of mutations in cryptococcus as a result of transposon mobilization [39,40], one can imagine that climate change could trigger positive feedback loops whereby increased temperatures trigger genetic changes that can enhance virulence, reduce drug susceptibility and generate diversity that can allow colonization of new ecologic niches.

**Figure 1.**
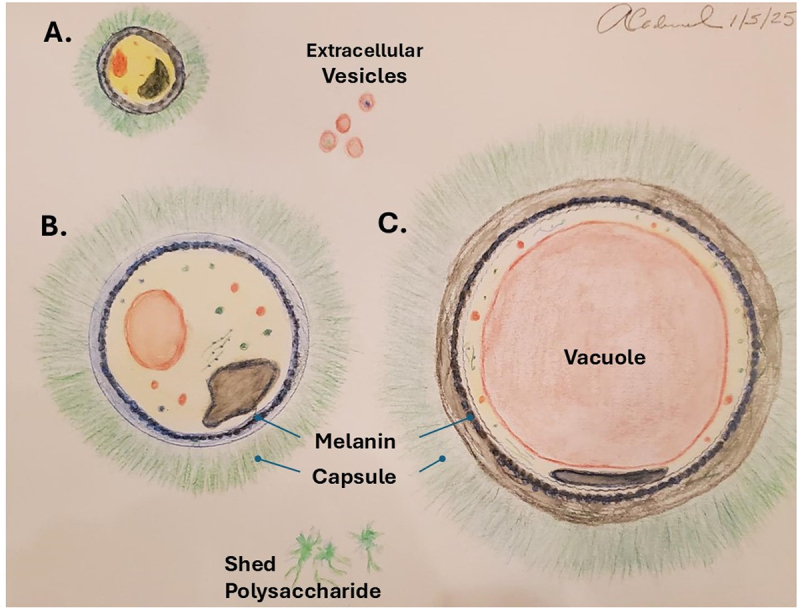
The anatomy of cryptococcal cells. A. small cell of the type that is sometimes observed in cryptococcal infection. B. typical cryptococcal cell growing in laboratory media. C. Giant or Titan cell, which is triggered by activation of several signaling pathways [43,44]. Melanin is deposited in the cell wall. Polysaccharide is shed into culture media or tissue, as are extracellular vesicles [45]. Watercolor by author.

Morphologically CN and CG cells are often described as “yeast like” to denote the fact that they are spherical or nearly so and usually reproduce by budding. CN cells usually range in size from 5–10 μm but can enlarge tremendously to form so-called Titan cells that can range up to 50–100 μm in diameter [41,42] (Figure 1). During infection, CN also generates very small cells ranging from 1–3 μm [42]. Small cells have not received much research attention but recently were implicated in extrapulmonary dissemination and consequently been named “seed” cells [46], suggesting a hitherto unrecognized importance in pathogenesis. Cryptococcal cell walls have more chitosan than chitin, which is the de-O-acetylated form of chitin, and is essential for cell wall integrity and virulence [47–50]. The predominance of chitosan in the cryptococcal cell wall may have evolved as defense against chitin degrading enzymes [51], which are found in certain hosts like plants and amoeba. Unlike yeast cells, CN cells are surrounded by a polysaccharide capsule that is usually invisible when cells are suspended in aqueous solutions because it is comprised primarily of water [52], and consequently, its refractive index is so close to water that it cannot be discerned by light microscopy. To visualize the capsule, cells are often suspended in India ink and the ink particles cannot penetrate the capsule, thus creating a visible halo in the capsular space. The capsule can also be visualized by adding an antibody that binds to the capsular polysaccharides. Antibody binding changes the refractive index of the capsule such that it can be visualized by light microscopy in a process known as capsular reaction (or ‘quellung effect), as has been described for encapsulated bacteria [53,54]. Recently, Percoll was used to visualize the capsule, which has significant advantages because it does not interfere with budding [55].

CN cells exhibit remarkable capacity to enlarge and become giant cells. These were occasionally noted during microscopic examination of infected tissues [42] but careful exploration of the phenomenon did not begin until 2010, when two independent laboratories simultaneously published their isolation and characterization results in the laboratory [56,57]. Now known as “Titan cells” [58], their occurrence shows a new variation in the story of fungal dimorphism, whereby CN cells increase spherically, rather than changing morphology from yeast to hyphae or vice versa. Given that these cells can reach 100 μm in diameter, their size is practically within the resolution of the human eye, which makes them macroscopic. The study of Titan cells is now a major endeavor in the cryptococcal field; however, in the years after their discovery, it was difficult to study them because they were observed only during infection. A major advance occurred in 2018 when three independent groups described the conditions for the generation of such cells in vitro [43,59,60]. As with the original reports on the discovery of Titan cells [56,57], the three groups coordinated their submissions for simultaneous publication, providing another example of the collaboration and comradeship that characterizes the cryptococcal research community. These reports showed that there are several different pathways for triggering the transition from regular yeast cells to Titan cells, suggesting redundancy in this morphological transition. Titan cell formation is regulated by the cAMP/PKA signaling pathway [61] and appears to be a result of continued mitosis without cell division, such that the cells become polyploid through a mechanism involving cyclin Cln1 and G2 arrest [62]. Consistent with the view that Titan cell formation is triggered by cell damage, its formation is associated with the accumulation of endogenous free radicals [63]. At this time, the best synthesis of the information available on Titan cell formation suggests that this transformation is a defensive mechanism for survival because such cells are so large that they cannot be ingested by amoeboid predators in the environment or macrophages. In this regard, it is noteworthy that exposure of CN to amoeba or macrophage lipids is a trigger for capsular enlargement, and one can imagine how sensing of danger by CN cells can turn on a program for gigantism whereby the likelihood of survival increases by achieving larger cell sizes.

CN strains are capable of phenotypic switching [64], a phenomenon whereby a fungal strain generates colony variants of differing morphology that can revert to the original morphology at a rate much higher than the species mutational rate [65]. Phenotypic switching is associated with changes in virulence and has been demonstrated to occur during experimental murine infection, whereby it was associated with changes in virulence and a lethal outcome [66], a phenomenon that may also occur in humans [67]. In this regard, phenotypic switching is associated with the generation of variants that are more likely to disseminate to the brain [68] and to affect virulence by affecting the host immune response [69], through effects on macrophage activation [70]. Although our understanding of the molecular mechanisms responsible for phenotypic switching remains incomplete, the allergen 1 gene (*ALL1*) was implicated in this phenomenon when it was downregulated in mucoid variants and deletion of the gene produced a very similar phenotype [71]. Loss of *ALL1* was associated with increased intracranial pressure and changes in polysaccharide structure, correlating with the production of mucoid colonies [72]. In recent years, several studies have shown additional links between morphological changes and virulence and have implicated epigenetic mechanisms responsible for some of the observed variation [73,74].

A consistent theme with CN is the remarkable phenotypic and genetic variability inherent to this fungal species. An early study of isolates of a type strain that was being studied in several laboratories showed that when these were compared, they had undergone significant phenotypic and chromosomal polymorphisms as a result of microevolution [75]. This result indicated that CN evolved in different laboratories and provided a cautionary tale when comparing interlaboratory findings involving similar experiments with the same strain. Serial isolates from patients belonging to the same strain manifest chromosomal polymorphisms that are consistent with microevolution during infection [76]. CN aneuploidy is common and contributes to drug resistance [77]. Chromosomal instability appears to be an intrinsic characteristic of CN and is regulated by the multi-protein striatin-interacting phosphatase and kinase (STRIPAK) complex [78]. This characteristic presumably provides a mechanism for the generation of rapid phenotypic variation that can provide adaptability for cryptococcal cells in diverse environments, including during animal infections.

CN grows well on most fungal media and can be easily adapted to minimal media consisting of glucose and a simple nitrogen source, such as glycine. Hence, this fungus has the metabolic capacity to produce all the compounds required for growth from simple sugars and amino acids. The fungus prefers an acidic milieu and grows best in the pH 4–5 range of [79]. Most strains tolerate temperatures of up to 40°C [80], which is permissive for survival at human core temperatures. Numerous genes and pathways have now been associated with thermal tolerance and adaptation to mammalian hosts, including Ccr4-mediated decay of ribosomal protein mRNAs [81], Set302 (a homolog of Set3 and a subunit of histone deacetylase complex) [82], and uncharacterized genes [83], such as TVF1 (thermotolerance and virulence-related factor 1) [84]. Similar to other microbes, CN was recently reported to have a viable but non-culturable phenotype, which appears to be a defense mechanism for survival in response to stressful conditions [85]. Such cells appear to be a mechanism by which CN can enter dormancy in vivo, a condition that can reactivate into disease if there is a change in the immunological status of the host [18]. Remarkably, dormancy can be maintained inside macrophages, but these cells can also reactivate dormant cryptococcal cells using a combination of extracellular vesicles and non-lytic exocytosis, implicating host cells in both the maintenance and reactivation of dormant infections [86].

### The life cycle

The *C. neoformans* species complex is an environmental fungus that occupies a variety of niches. Fungal cells come in two mating types known as “**a**” and α, and can reproduce asexually (budding) or by the production of basidiospores after mating. *Cryptococcus* spp. are also capable of same-sex mating to produce basidiospores [87,88]. From the viewpoint of mammalian virulence, basidiospore production is a critically important event in the cryptococcal life cycle, as these structures are likely to be infectious propagules [89]. CN has traditionally been associated with pigeon excreta, whereas *C. gattii* has been found to be associated with a variety of tree species, including eucalyptus. However, in recent years, there have been many reports of CN isolation from trees, suggesting that both species complexes share arboreal niches [90,91]. In fact, there is an emerging consensus in the field that cryptococcal residence in plants and soils reflects a natural habitat that is critical for its life cycle given that plants can promote mating, generation of basidiospores and the dispersal of the fungus [92–94]. Hence, it is possible that the frequent isolation of CN from sites contaminated with pigeon excreta, which often follows from the urbanization of pigeons, is a reflection of the capacity of this fungus to use pigeon excreta as a growth medium rather than its original natural habitat []. In this regard CN can utilize uric acid, which is a product of avian nitrogen metabolism that is abundant in pigeon excreta as a nitrogen source [95] and the fungus has all the genes necessary to break down this compound [96]. Hence, the common association of CN with pigeon excreta could be more reflective of the fortuitous coincidence of the anthropomorphic global dispersal of pigeons in historical times combined with metabolic machinery for uric acid utilization rather than their original ecologic niche. Knowing the natural habitat is important for understanding the mechanisms of virulence, which are almost certainly accidental adaptations of strategies used for environmental survival in a phenomenon we have called “accidental virulence” [97]. In other words, CN does not require an animal host for any part of its life cycle, and consequently, the mechanisms of virulence must reflect the application of environmental survival strategies for survival during infection.

The fact that the CN can reproduce both asexually and sexually is reflected in studies of its population structure. The earliest insight into the population structure came from isozyme analysis, which suggested that it was clonal [98]. When genetic markers were developed, they were applied to clinical isolates from New York City and Brazil, suggesting clonal population structure [99,100]. Subsequent studies comparing clinical and environmental strains confirmed the notion that the population structure was predominantly clonal but also found evidence for genetic recombination [101], thus providing firm evidence for both sexual and asexual reproduction. In fact, some evidence of genetic recombination appears to originate from same-sex mating in nature [102]. The ability to reproduce clonally and sexually provides the potential for a tremendous amount of genetic diversity in *Cryptococcus* spp. complexes. As noted above, the cryptococcal genome is plastic, and environmental strains may be more prone to recombinant DNA changes than clinical strains [103,104]. Passage of cryptococcal strains into humans and mice is sufficient to produce karyotype changes, genomic mutations, and rearrangements [105–107]. Growth at mammalian temperatures is sufficient to mobilize genetic elements that can introduce genomic changes and promote rapid adaptation during infection [39,40]. Hence, for CN,residence in the ecologic niche defined by a mammalian host provides a major stress that is associated with genetic changes, the possibility for immune selection and a chance for adaptation to the new conditions. Host death would provide CN with the possibility of returning to the environment and those changes would incur fitness costs or benefits depending on the new conditions.

### Infection, disease and epidemiology

Cryptococcosis is caused by CN and CG species complexes, which are collectively responsible for more than 100,000 deaths worldwide each year [108]. Among these, most cases are caused by CN, which has a predilection for immunocompromised patients. In addition to causing cryptococcosis, CN may contribute to some cases of fungal asthma given its propensity to cause chronic lung infections that can predispose to airway hyperresponsiveness [109]. In 2022, the World Health Organization designated CN as a critical priority pathogen given its prevalence as an agent of disease, and the mortality and morbidity associated with cryptococcosis [110].

Given the ubiquity of CN in urban environments, human exposure to this fungus is likely common. Consistent with this assumption, serological studies have shown that antibodies to CN antigens are common among both healthy individuals and those at risk for disease [111–113]. A study in New York City, a city where large flocks of pigeons abound [114], found that babies were born with maternal antibodies, which declined in the first years of life and then increased after age 2, consistent with childhood infection [][115]. To put these results in perspective, it is worth noting that a serological study of New Yorkers found no adult seronegative individuals [115]. This implies that CN infection of sufficient intensity to elicit an adaptive humoral response was universal among New York City dwellers who co-inhabit a city with large pigeon flocks that frequently contain heavily contaminated sites [,^114^,^116^]. Among the pediatric individuals studied, one had cryptococcal antigen in the serum at the time the child had symptoms requiring a clinic visit, raising the possibility that the initial bout of CN may be one of the diseases of childhood that is currently without an etiology [,^115^]. While serology suggests that the infection is acquired in childhood, pediatric cryptococcosis is extremely rare. Overall, the serology data indicate that while CN infection is quite common and may be universal in certain cities, the disease is rare. Whether the initial infection is asymptomatic or if its symptoms are not recognized as a discrete clinical entity and consequently not diagnosed is not known.

Cryptococcosis was first described in 1895 by Sanfelice in Italy and Busse and Buschke in Germany [25] but was an exceedingly rare disease in the first half of the 20^th^ century. CN was reported as a pathogenic microbe in the late 1890s [25,117], toward the end of the years that marked the germ-theory revolution. The relative lateness in recognition as a cause of human disease compared to bacterial pathogens was undoubtedly a reflection of the fact that cases of cryptococcosis were few and far between. The rarity of CN-related disease among immunocompetent individuals is a strong testament to the high effectiveness of the mammalian immune response in the presence of this fungus and in preventing disease. In fact, by the 1950s, only about 300 cryptococcosis cases had been described in the literature worldwide. However, by 1950, there were indications that the prevalence of cryptococcosis had increased worldwide. Littman and Borok noted that there were only eight cases in New York City from to 1950–1953 but 25 from to 1954–1958. The 1950s was a time of tremendous progress in medicine, with the development of immunosuppressive therapies in the form of corticosteroids and the first effective chemotherapy for cancer. However, this progress came at the expense of immunity, and as the number of immunosuppressed individuals increased, cases of cryptococcosis increased. In the subsequent decades, the prevalence of the disease continued to increase slowly until the 1980s when cryptococcosis was recognized as a complication of AIDS. By 1991, there were over 1200 cases of cryptococcosis in New York City alone, implying a 500-fold increase in the prevalence of the disease from the early 1950s, with most cases occurring in patients with AIDS. The introduction of effective antiretroviral therapy in the 1990s restored immunity in patients with advanced HIV infection, which in turn reduced their susceptibility to CN.

In the early 21^st^ century, the epicenter of the HIV pandemic had moved to sub-Saharan Africa, where millions of individuals with AIDS were at risk for cryptococcosis. This resulted in a major increase in worldwide cases; in 2009, the global burden of disease was estimated to be approximately one million cases with over 600,000 deaths per year, with the majority of cases occurring in Africa [118]. As access to antiretroviral therapy has become more available worldwide, including in resource-poor regions, the prevalence of AIDS-related cryptococcosis has declined; in 2020, it was estimated that the number of annual deaths had declined to approximately 152,000 [119]. In recent years, cryptococcosis has been recognized as a complication of COVID-19 [120], although the exact prevalence is unknown because most cases remain unreported.

CN has a predisposition to cause diseases in males [24]. The increased susceptibility of males to cryptococcosis has been noted for decades, but the underlying mechanism is poorly understood [121]. Evidence suggests that this explanation may be multifactorial. Macrophages from human females were more active in phagocytosing CN than those from males, but macrophages from males had a higher intracellular fungal burden [122], suggesting a reduced ability to control intracellular proliferation. Incubation of CN with peripheral blood mononuclear cells revealed from males greater fungal proliferation and reduced T cell percentages relative to the same experiment with cells from females [123]. When CN is exposed to sex hormones, it melanizes faster in the presence of testosterone than estradiol [124], and faster melanization has been associated with increased virulence [125].

The epidemiology of cryptococcosis reflects the immunological health of the human population. In the early 20^th^ century, when most individuals in the population had intact immunity, cryptococcosis was an extremely rare disease that probably occurred only in individuals with inborn metabolic errors that weakened immunity. However, by the mid-20^th^ century, the prevalence of disease began to rise as autoimmune and neoplastic diseases were treated with regimens that weakened immunity, leaving individuals at risk for fungal diseases. The cataclysm of the AIDS pandemic brought the prevalence of cryptococcosis to epidemic levels that have fortunately abated as therapies for HIV have improved. Going forward, it is likely that the disease will remain stubbornly common, since the number of individuals surviving cancer and receiving organ transplants continues to increase. From this perspective, the number of cryptococcosis cases is a barometer for the number of immunocompromised patients in the population.

### The origin of cryptococcal virulence

CN infections in humans are acquired from the environment. This is because the fungus is not part of the host microbiome but is found in the environment, often near human dwellings. In the early 1990s, an analysis of clinical and environmental isolates using molecular probes showed that these were indistinguishable [116], further supporting an environmental origin for human infection. The first well-documented point source for human infection came from a case report involving a pet cockatoo where the patient isolate was indistinguishable from CN from bird excreta [126]. Two decades later, a follow-up analysis of the patient and cockatoo isolate using DNA sequencing confirmed the close genetic identity of these samples and identified several mutations associated with human passage [107]. Passage of the CN isolate from cockatoo stool in mice led to genomic changes in the same loci as those mutated during human passage, implying that adaptation to the environment in the mammalian host was associated with the selection of certain mutations [107]. This observation could help explain the finding that environmental strains are often hypovirulent relative to clinical strains [127], and implies that human passage can change the infecting strains such that they are not identical to the environmental parent.

The comparison of CN strains to their close relative nonpathogenic species in the genus Cryptococcus provides additional insight into its virulence. In 2001, a survey of heterobasidiomycetous yeasts comparing CN to other species revealed that only CN was endowed with the capacity for growth at 37 C, possessed a capsule, and had the CNLAC1 gene that catalyzes the synthesis of melanin [128]. More recently, a comparative analysis of closely related species in the *Cryptococcus* and *Kwoniella* genera revealed that pathogenic cryptococcal species possess a gene encoding a putative D-lactate dehydrogenase that was not found in nonpathogenic close relatives [129]. Interestingly, D-lactate dehydrogenase may have been acquired by lateral gene transfer from *Aspergillus* spp. and appears to have an ancient bacterial origin [129]. Although the role of this gene in virulence is currently unknown, it represents part of an emerging genomic signature for pathogenic cryptococcal species, consistent with the notion that the capacity for mammalian virulence could have originated from a confluence of events that in aggregate endowed CN with the ability to resist clearance by immunological mechanisms.

A fascinating aspect of cryptococcal pathogenesis is that cells differ in virulence depending on their chronological age [130]. Cryptococcal infections can be chronic and slow progressing, giving cells in tissue the opportunity to age in the host. As cells age, their resistance to oxidative killing, macrophage intracellular killing, and antifungal agents increases [130]. This resistance leads to the selection of older cells, resulting in positive feedback for increased virulence over time. The lifespan of cryptococcal cells is modulated by sirtuin 2 (SIR2) with SIR2 deficient cells having a reduced lifespan [131], which has significant implications for the development of drugs targeting this pathway. Cellular aging is associated with changes in the cell wall [132], ABC efflux pumps that affect fluconazole susceptibility [133] and mitochondrial function [134] creating a situation where the virulence potential and antifungal susceptibility of CN cells in tissues differ as a function of time and undoubtedly complicate the capacity of immune responses and drug therapy to eradicate the infection. Notably, the phenomenon of cryptococcal aging and virulence was discovered during investigations of phenotypic switching [135], illustrating the continuity of two processes that generate cellular diversity.

When considering the origin of cryptococcal virulence, one must face two questions: 1) Why does a soil organism with no need for animal infection in its reproductive cycle have the capacity for virulence? 2) How does CN have the capacity to infect and cause diseases in diverse hosts such as mammals [24], insects [136], fish [137], reptiles [138], birds [139] amphibians [140], and plants [141]? One can add that CN can infect unicellular hosts such as amoeba and paramecia after the yeast is ingested. Hence, CN is a generalist type of pathogenic microbe endowed with tools to survive in diverse hosts, which present very different types of environments. These tools, in turn, must have been selected for survival in the CN natural habitat, which thus far has been identified as soils, trees, and tree hollows. Hence, when considering the origin of virulence for CN, it is necessary to consider the selection pressures that operate in the natural environment.

### CN interactions with unicellular eukaryotic predators including macrophages.

The capacity of amoebas to prey on CN spp. was first noted by Castellani in the 1930s [142]. Since then, the amoeba-fungus interaction has been periodically studied with the consistent finding that various species of amoeba ingest and kill *Cryptococcus* ssp [143,144]. Beginning in the late 1970s, Bulmer et al. carried out detailed studies of the interaction of amoeba and CN and they noted that *Acanthamoeba polyphaga* avidly ingested yeast forms but not hyphal cells [145,146]. This group also provided evidence that amoebae preyed on CN in soils contaminated with pigeon excreta, thus implicating them as an important biotic control factor in the environment [147]. Analysis of the functions of the CN capsule, melanin, and phospholipase in the interaction with amoebae revealed that these virulence factors for mammalian pathogenicity protected the fungus against amoeba. This observation, together with the finding that CN-amoeba and CN-amoeba macrophages were similar, led to the proposal that the capacity for virulence in CN originated from the eons of selection in the environment by amoeboid predators [][148]. The connection between predation by eukaryotic unicellular organisms and CN virulence was supported by the demonstration that the passage of a cryptococcal strain that had lost virulence in the presence of *Dictyostelium discoideum* restored virulence [149]. CN-amoeba interaction demonstrates many of the same phenomena observed in macrophages, such as non-lytic exocytosis [150,151] and cryptococcal capsular enlargement [152]. However, amoebae are not the only predators of CN in the soil. Paramecia [153] and *C. elegans* [154] have both been demonstrated to engorge themselves with yeast cells when fed CN under laboratory conditions, and both mites are sow bugs that can consume this fungus [147].

When considering CN-amoeba interactions in the context of the evolution of fungal virulence, it is important to take a nuanced view and acknowledge that not all outcomes will result in increased virulence. Under laboratory conditions, the passage of CN with amoeba can result in increased [149,155–157], unchanged [158] or decreased [146,159] animal virulence. This inconsistency in outcome is not surprising since there are likely to be multiple solutions to the problem of cryptococcal survival in amoeba, and not all of these solutions would necessarily result in increased fitness for growth in macrophages or virulence in mice. Hence, small differences in CN-amoeba interaction could translate into large differences in the outcome of CN infection in mice, as the process of selection and adaptation may have stochastic variables. In fact, this principle was evident from the earliest studies of Bulmer et al., who showed that CN pseudohyphal forms were more resistant to amoeba predation but less pathogenic in mice [146]. The converse is also likely to apply in that not all cryptococcal solutions for increased growth in macrophages and animal virulence would necessarily result in increased fitness for amoeba. When isolates from localized or systemic human infections were co-incubated with amoeba, the latter were more cytotoxic to protozoan cells [160]. When considered as a group, these studies suggest that the CN-amoeba interaction can be a powerful selection system for both entities, and that the outcome of the interaction can have variable and possibly unpredictable effects on CN virulence in animal hosts.

Once an amoeboid predator, such as *Acanthamoeba castellani* ingests a cryptococcal cell, it acquires an intracellular infection that can lead to food for, or death of, the host cell. In recent years, several studies have provided detailed molecular dissection of the changes associated with amoeba and fungal cell survival. *A. castellani* appears to inhibit CN intracellularly by depriving it of zinc, thus providing a form of unicellular nutritional immunity [161]. CN has evolved resistance mechanisms to amoeba predation, as the capsule is antiphagocytic and melanin protects the fungal cell against killing one ingested. There may be genetic mechanisms in place to enhance fungal variability as a defense mechanism against ameboid predators. Such a mechanism was suggested by the discovery of a genomic locus encoding the transcription factor Bzp4 that confers fungal phenotypic variation that, when deleted, increases susceptibility to amoeba predation [162]. Since CN in soils would likely face many ameboid predator species, the fungus must have nonspecific defense mechanisms and the ability to generate variability could provide a survival advantage, as some phenotypes would be more resistant to some amoeba species than others, increasing the likelihood of survival.

The “Ameboid predator-fungal animal virulence” hypothesis posits that interactions between fungi and environmental ameboid predators can result in the selection of fungal traits that give the fungus the ability to cause disease in animals [163]. This hypothesis emerged from observations with CN [,^148^], where the capacity for animal virulence was an emergent property from unrelated selection pressures, leading to the concept of “accidental virulence” [97]. When considering the outcome of experimental CN-amoeba interactions, it is noteworthy that the hypothesis does not posit that all outcomes would result in increased virulence. As noted above, there are reports that fungal passage in amoeba can both increase and decrease animal virulence. Experiments passing CN in amoeba showed that not all repetitions resulted in the same outcome. Hilbert reported that when CN was passaged in amoeba and macrophages, some outcomes produced fungal selection that was better for survival in these cells, whereas others had no effect [159]. Hence, the outcome of the CN-amoeba interaction is variable, unpredictable, and has some elements of stochasticity.

**Figure 2.**
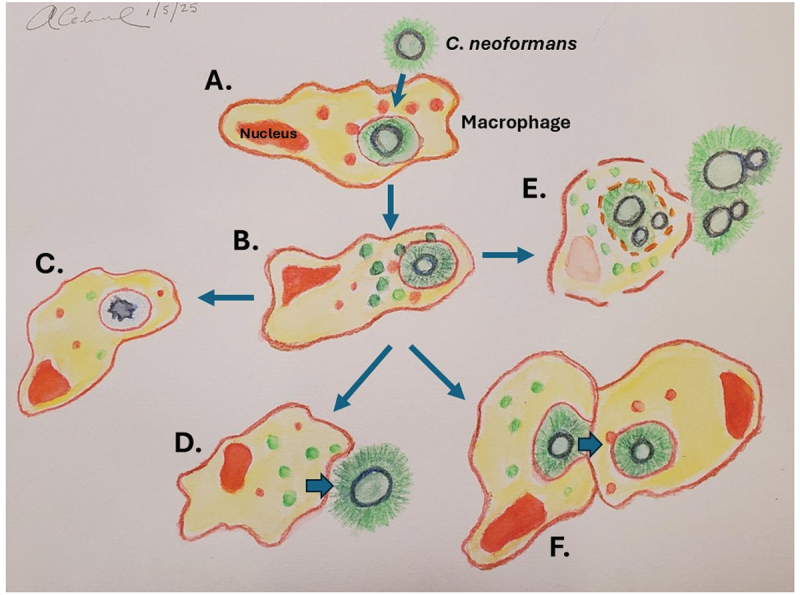
Diagram of interaction of CN with macrophages. A. macrophage ingests CN, a process that is inefficient in the absence of antibody or complement opsonins. B. Intracellular residence is associated with the production of polysaccharide laden vesicles that accumulate in the cytoplasm [169]. In some macrophages fungal cells are killed by host cell antifungal activity (panel C) while in other macrophages continued intracellular fungal replication can lead macrophages lysis. Panel D illustrates CN non-lytic exocytosis whereby the fungal cell exits the macrophage [172,175]. Panel E illustrates macrophage lysis with release of fungal cells. Panel F illustrates transfer of CN between two macrophages [173,174], in a process that has been named dragotcytosis [176]. Watercolor by author.

Many of the facets of CN interactions with amoeba are recapitulated during its interaction with macrophages. Until the late 1990s, CN was considered an extracellular pathogen. This is because CN lesions often showed masses of extracellular organisms in the tissue. The large polysaccharide capsule reinforced this impression given its strong antiphagocytic properties and the fact that encapsulated bacterial pathogens, such as *Streptococcus pneumoniae*, were considered extracellular pathogens. Although tissue histology often revealed intracellular CN-in macrophages, these were assumed to be ingested cells that were probably dead. Consistent with this impression were in vitro observations that macrophages could kill ingested CN [164,165]. There have also been reports of CN growth inside macrophages [166,167] in vitro, but the connection to possible intracellular pathogenesis had not been established. In addition, there are various hints that CN is capable of intracellular survival, including its ability to establish latency in granulomas and the fact that increased susceptibility is correlated with defects in T cell immunity. In 2000, an ultrastructural study of pulmonary infection in mice showed that the budding index for intracellular CN was approximately five times greater than that for extracellular cells, consistent with and indicative of intracellular growth in vivo [168]. Subsequent studies established that CN had a distinctive intracellular replication strategy that included the production of intracellular polysaccharide-laden vesicles with compromise of the phagosomal membrane, as well as unique events such as non-lytic exocytosis and cell-to-cell transfer between macrophages [169]. For CG adaptation, survival in the intracellular environment is associated with morphological changes in the mitochondria that appear to result from oxidative stress [170]. Two novel processes associated with CN intracellular pathogenesis are the phenomenon of non-lytic exocytosis, whereby yeast cells escape macrophages without lysis of the host cell [171,172] and macrophage-to-macrophage yeast cell transfer [173,174] (Figure 2). The latter phenomenon was subsequently shown to be a sequential non-lytic exocytosis event followed by phagocytosis by another macrophage [176]. Intracellular residency with macrophage metabolic reprogramming appears to be involved in dormancy, whereby CN infection can remain quiescent in certain hosts for prolonged periods [177]. The discovery of a sophisticated intracellular replication strategy brought the conundrum of explaining why a soil organism with no requirement for animal virulence would have the capacity to survive in mammalian macrophages. Furthermore, a comparison of the interactions between genetically distant CN strains and macrophages revealed that conservation of the intracellular pathogenesis strategy led to the inference that it dates back to the Cretaceous [178]. The combination of sophistication, ancientness, and similarities between amoebas and macrophages supports the notion that the CN intracellular pathogenic strategy evolved long ago as a mechanism for survival upon ingestion by amoeboid predators. However, given its ancientness, it is also possible that the CN intracellular pathogenic strategy was adopted for survival in a now-extinct animal host and/or this fungus has cycled through many animal species in the past. According to this view, cycling through animal hosts would require survival in that host, which could lead to the selection of some phenotypes that could have survival advantages against ameboid predators when the animal died, and the fungus returned to the soil.

### The determinants of virulence

CN has a suite of properties and qualities that give it the capacity for virulence. CN is unusual among pathogenic fungi in that it has classical virulence factors that are required for virulence but are dispensable for growth. The major virulence factors of CN are the polysaccharide capsule, laccase and its product, melanin, and the enzymes urease and phospholipase. For each of these features, it is possible to generate mutants that lack the trait, manifest reduced virulence, and grow normally under laboratory conditions. In addition to its classical virulence factors, numerous other traits are also associated with CN virulence. Clinical strains are disproportionately of the alpha mating type [179], which is more virulent.

CN does not produce any known toxin. Each virulence factor plays environmental roles and functions during infection by protecting the fungus against immune mechanisms. Table 1 lists the virulence factors with putative functions in the environment and during virulence, which were inferred from laboratory studies. Some of the mechanisms by which CN virulence factors undermine immunity appear to be emergent properties of their interaction with immune cells. For example, shed capsular polysaccharides, which play a role in biofilm formation, accumulate in tissues and interfere with various immune functions, resulting in ineffective local inflammatory responses. Similarly, urease, which has a nutritional role in allowing the utilization of urea as a nitrogen source, facilitates brain invasion and alters the type of inflammatory response, possibly through local pH effects. Emergence appears to play a role in the regulation of virulence factors. In this regard, urease activity promotes melanization through pH effects, whereas melanization reduces urease activity by reducing the urease-containing release of extracellular vesicles [206]. In addition, melanization interferes with capsular formation by chelating Ca2+ ions, which are essential for assembling the outer layer of this structure [207].

**Table 1.** Virulence factor function in environment and pathogenesis.

Virulence factor	Environmental Role	Pathogenesis Role
Capsule	Anti-phagocytic [208] Desiccation protection [11] Biofilm formation [180] Buoyancy [181]	Antiphagocytic Immune modulator [182,183] Biofilm formation [180]
Melanin/Laccase	Protection against amoeba [208] Mechanical strength [184] UV protection [185] Protection against cold/heat [186] Heavy metal protection [187]	Oxidant protection [188] Antifungal drug protection [189] Prostaglandin synthesis [190]
Urease	Nutrition, Nitrogen acquisition [79]	Phagosome alkalinization [79] Brain invasion [191]
Phospholipase b	Nutritional Cell wall integrity [192]	Phagosome damage [193] Brain survival [194] Adhesion to lung cells [195] Extrapulmonary dissemination [196] Lipid metabolism [197]
Phospholipase c	Plasma membrane stability [198]	Regulation virulence factors [199] Survival in macrophage [200]
Protease	Nutritional	Tissue damage [201]
D-mannitol	Stress resistance [202]	Capsule enlargement [203] Reduced killing [204] Increased intracranial pressure [205]

When CN invades human tissues, it can co-opt compounds for increased virulence. The brain is a rich source of catecholamines for melanin [209]. Urea is abundant in tissues because of host nitrogen metabolism, and this molecule is hydrolyzed into carbon dioxide and ammonia by CN urease, with the latter altering phagosomal pH to promote survival in macrophages [79] and aid in brain invasion [191]. Inositol in brain tissue can be used as a carbon source by CN [210], and its presence triggers capsule synthesis, making fungal cells more resistant to host phagocytic cells [211]. Hence, physiological tissue components such as catecholamines, urea, and inositol are metabolized by CN to create a positive feedback loop that interferes with host immune clearance mechanisms, such that once this fungus gains a foothold during infection, conditions at the site can increase its virulence.

When approaching the tools for virulence of CN, it is important to move beyond classical virulence factors and recognize that during mammalian infection, the yeast must adapt to a hostile environment, which requires a complex suite of stress responses and metabolic changes. Hence, numerous signaling and metabolic pathways are required for virulence, which is a very active area of research [212,213]. Infection involves establishing a niche within a host, which requires surviving immune attack, higher temperatures, and obtaining vital nutrients, despite nutritional immune mechanisms that seek to deny essential elements such as iron. Hence, mammalian infection can be viewed as a highly stressful environment for CN that requires multiple metabolic adaptations. These adaptations involve a multitude of processes including protein ubiquitination [214], lipid modification of proteins [215], calcium homeostasis [216], Copper metabolism [217], autophagy [218], zinc availability [219], iron metabolism [220], among others. Mitochondria have emerged as critical organelles for virulence that function by detoxifying the fungicidal molecules produced by immune cells [221,222]. Changes in metabolism appear to be the key virulence factors [223]. In addition, mitochondria also facilitate virulence by providing the energy required to survive infection, and a mutation in NADPH hydrogenase, which increases cellular ATP, is associated with increased virulence [224]. Mitochondria are also involved in iron acquisition from heme, which is the most abundant source of iron in infected animals [225,226]. Consistent with these mitochondrial effects, the hypervirulent lineage associated with the Vancouver *C. gattii* outbreak manifested the upregulation of many mitochondrial genes [227].

### The mechanisms of host damage

**Figure 3.**
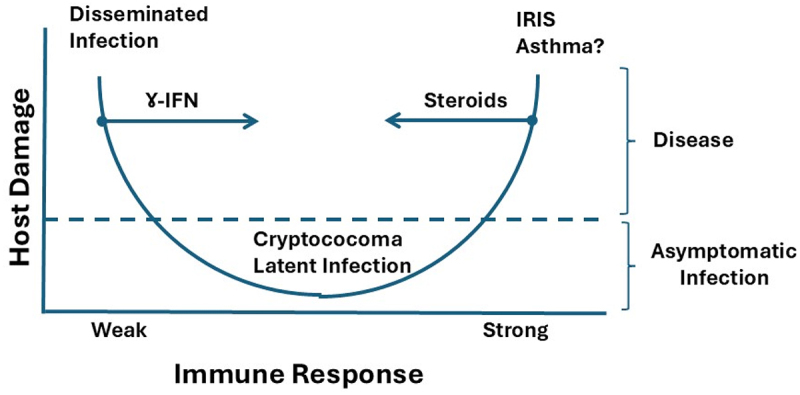
Pathogenesis of CN-related disease as viewed by the damage-response framework of microbial pathogenesis [228]. According to this theory disease occur when damage is sufficient to alter homeostasis, and this occurs in situations of inappropriately weak or strong immune responses. Most cryptococcal infection are asymptomatic, leading to clearance or persistence in a latent state that can occur in cryptococcomas. In hosts with weak immune responses CN infection can disseminate producing meningoencephalitis. In hosts with strong immune responses the interaction with CN has been proposed to trigger allergic responses that can predispose to hyperreactive airway disease. In patients with AIDS who survive cryptococcosis and recover immunity as a result of antiretroviral therapy immune recognition of residual fungal products in tissue can cause the immune reconstitution syndrome (or IRIS). The arrows represent potential immunotherapy to reduce damage. In immunocompromised hosts such as patients with AIDS and cryptococcosis the addition of interferon-gamma to therapy produced encouraging results [229] while corticosteroids have been used to treat IRIS.

The most frequent presentation of cryptococcosis in humans is cryptococcal meningoencephalitis, which is uniformly fatal if not treated with antifungal agents. Since CN does not produce any known toxins, host damage is believed to be the result of mechanical processes such as high intracerebral pressure and/or inflammatory response. CN is a prototypical pathogen for the damage-response concept of microbial pathogenesis, whereby host damage tends to occur at the horns of the parabola, which corresponds to insufficient and exuberant inflammatory responses (Figure 3). AIDS-related cryptococcosis is associated with a high fungal burden, which can lead to life-threatening increases in intracerebral pressure due to tissue edema and/or blockage of cerebrospinal fluid drainage. Older studies have shown that capsular polysaccharides can directly cause cellular edema [230–233] and that there is an association between increased intracerebral pressure and CN capsule size [234]. These observations, combined with the fact that during CN meningoencephalitis there is a capsular polysaccharide through the brain parenchyma [235], suggest a possible mechanism for cerebral edema. In addition to tissue edema formation, CN cells and shed polysaccharides can cause obstruction of CSF outflow, which often requires shunt placement to relieve intracranial pressure [236]. Polysaccharide release in tissue has an environmental correlation in that this phenomenon is involved in biofilm formation [237]. Recently, ammonia generated by cryptococcal urease was implicated in virulence [238]. In patients with AIDS-related cryptococcosis, the advent of antiretroviral therapy led to the recognition of immune reconstitution inflammatory syndrome (IRIS), which causes a tissue-damaging inflammatory response to residual fungal antigens and cells. In addition, there is circumstantial evidence that cryptococcal infections can promote hyper-reactive airway diseases [239]. Hence, the mechanisms of CN tissue damage include physical (pressure, obstruction), inflammatory, and possibly hypersensitive reactions.

### The effectiveness of the immune response

At the height of the AIDS pandemic before the development of antiretroviral therapy, approximately 10% of all affected individuals developed cryptococcosis [24], which was essentially incurable, since even those who responded to initial therapy required life-long antifungal therapy. However, it is worth noting that even among severely immunosuppressed individuals with AIDS, only a minority developed cryptococcosis despite presumed continued exposure to this fungus from environmental sources, suggesting that residual immunity was sufficient to prevent the disease, even with severe depletion of CD4 T cells. On the other hand, some cases of cryptococcosis always occur among individuals with no obvious immune deficiency. In recent years, an explanation for cases of cryptococcosis among so-called immunocompetent individuals who were otherwise well has emerged, as modern medicine has uncovered subtle immunological defects. For example, the development of anti-granulocyte-macrophage colony-stimulating factor autoantibodies is associated with sporadic cases of human cryptococcosis in apparently normal individuals [240]. One can anticipate that, as more information becomes available about human genetics, more associations with cryptococcosis will follow. Continuing with our ecological theme, CN infection Immunocompetent and immunosuppressed hosts provide very different ecological sites for CN, with the former including a challenging granulomatous responses that can inhibit fungal growth with the latter often involving little of no inflammation [241].

The fact that immunologically intact hosts are highly resistant to CN-related diseases despite serological evidence for frequent exposure and infection implies that human immune mechanisms are highly effective at preventing CN invasion of host tissues. Information on the components of the immune system that are important for controlling CN infection can be deduced from the associations between specific immunological deficits and increased susceptibility to cryptococcosis. The fact that advanced HIV infection, steroid use, and antibody therapies based on blocking tumor necrosis each impairs cellular immune responses implies the importance of this immune arm in controlling infection. Hence, it is reasonable to conclude that cell-mediated immunity is a major host defense mechanism against CN. This conclusion is supported by extensive experimental work showing that T cells are critical for effective immunity [242,243]. The primary tissue defense mechanism against CN is granuloma formation, and T cells are essential for macrophage function. In contrast, defects in humoral immunity are not generally associated with an increased risk of cryptococcosis, although occasional case reports have shown that some individuals with hypogammaglobulinemia are vulnerable to CN-related diseases [244,245]. The general lack of association between cryptococcosis and humoral immunity defects is peculiar, given that CN is an encapsulated pathogen and that patients with antibody deficiencies are highly susceptible to disease from encapsulated bacteria such as pneumococcus. However, qualitative and quantitative defects in antibody immunity have been associated with increased susceptibility to CN in AIDS patients [111,246]. The lack of association between human cases of cryptococcosis and defective antibody immunity could simply reflect redundancy in immune mechanisms rather than a lack of importance for humoral immunity. In this regard, analysis of the serum antibody response to CN capsular polysaccharides in patients with AIDS showed deficits that could contribute to their susceptibility [246]. Laboratory studies have shown that capsule-binding antibodies are powerful opsonins for CN [247,248] and passive antibodies have been shown to be protective in mice [249–251]. Furthermore, antibody binding to the capsule can modulate cryptococcal metabolism and antifungal susceptibility [252], and some isotypes such as IgM appear to prevent titan cell formation [253]. Hence, the best interpretation of the available immunological data is that innate and adaptive immunity (both CMI- and antibody-mediated) all contribute to the remarkable human resistance to cryptococcosis.

### The prospect of vaccination

From an ecological perspective vaccines are agents that deny CN access to the host ecological niche by eliciting immune responses that prevent the establishment of infection and/or reduce infection-associated damage. Several independent groups have shown that it is possible to elicit protective immunity in experimental animals using various vaccine approaches that elicit cell-mediated immunity, humoral immunity, or both. Numerous antigens elicit protective responses [21]. Such experimental vaccines have included attenuated strains [254,255], killed cells [256,257], and component formulations such as polysaccharide-protein conjugates [258,259], extracellular vesicles [260], glucan nanoparticles [261], and most recently mRNA lipid nanoparticles encoding cryptococcal antigens [262]. Vaccination with recombinant cryptococcal antigens, such as polysaccharide deacetylases [263] and aspartyl protease [264], induces protective responses in mice. A particularly innovative approach is the construction of a cryptococcal strain that expresses interferon gamma, which elicits protective immunity [265]. Hence, there is no shortage of vaccine candidates for preventing cryptococcosis and the efficacy of vaccines can be enhanced with adjuvants [266–268]. The list of successful formulations that elicit protective immunity provides ample evidence for the notion that vaccines can be designed for the prevention of cryptococcosis, and that protection can be achieved by eliciting cell-mediated and/or antibody immunity. Furthermore, such vaccines could have therapeutic uses, given that the course of the disease is prolonged and associated with inadequate natural immune responses. The wealth of vaccine candidates and approaches available suggests that it is relatively easy to elicit protective immunity against this fungus. Consequently, the problem with prospects for vaccination against CN is less scientific and more in the realm of clinical testing and the economics of vaccine development (for an excellent essay on the challenge of vaccine development for cryptococcosis, see [269]). With regards to clinical testing, demonstrating efficacy is challenging because cryptococcosis is a sporadic disease even among susceptible individuals, and such trials would require enrolling a large number of participants to measure a meaningful effect. There is also a biological problem that any vaccine for cryptococcosis would have to elicit protective immunity in an immunocompromised group of individuals. Although immunosuppression poses a significant hurdle for vaccine design, it is noteworthy that other vaccines, such as those against varicella zoster virus and pneumococcus, are currently successfully used in immunocompromised individuals, implying that impaired immunity may be a hurdle but not a barrier. Hence, the prospect of vaccination against cryptococcosis is encouraged by the realization that such vaccines are possible while tempered by the difficulties inherent in clinical testing, the cost of clinical trials, and uncertainty about whether the size of the market would support their commercial production. However, it is possible that future conditions will increase the need to reduce obstacles. For example, if CN infection in childhood is firmly associated with subsequent diseases such as asthma, then market considerations would support development given the problem of asthma, as has happened with other vaccines such as the varicella vaccine that was initially developed for immunosuppressed children but is now marketed to all older adults. One issue seldom discussed in vaccination approaches is the intended purpose of the vaccine in the context of environmental acquisition versus reactivation. If infection is caused by basidiospores, prevention of infection may require a vaccine targeting spore antigens with the caveat that these rapidly germinate, such that the invasive form is the yeast. Therefore, targeting yeast cells may be sufficient to prevent infection. However, if the disease is caused by the reactivation of long dormant cells in granulomas, then it is likely that an immune response to cryptococcal antigens has already occurred during granuloma formation, and the purpose of a vaccine would be to enhance existing immunity to prevent disease. Hence, although great progress has been made in defining antigens that elicit protective immunity against experimental infection in animal models, uncertainties about the pathogenesis of human disease, ranging from the nature of the infectious propagule to the state of infection before the appearance of disease, provide additional hurdles in vaccine development.

### Diagnostic tools

Continuing with our ecological perspective, diagnostic tools provide the means of establishing the presence and location of CN in the ecologic niche defined by an infected host. The diagnosis of cryptococcosis is aided by the existence of sensitive and specific tools in the form of culture, antigen detection, and most recently, nucleic acid amplification (NAAT) tests. Given the availability of these diagnostic tools, the biggest hurdle for the early diagnosis of cryptococcosis is that physicians need to consider this disease early in their diagnostic approach to the patient. Many cases of cryptococcosis progress slowly with subtle and nonspecific symptoms, leading to a late diagnosis. In fact, there is arguably a perverse relationship between the relative ease of diagnosis compared to other fungal diseases and the clinical delays in diagnosis, since the fungus can proliferate to produce a large tissue burden before symptoms reach the point of triggering a major diagnostic workup. The large fungal burden facilitates diagnosis by providing plenty of cells for culture, copious cryptococcal antigens, and DNA for antigen tests and nucleic acid amplification techniques, respectively. Although culture has historically been the gold standard for the diagnosis of cryptococcosis, this disease has arguably the most successful antigen detection assay in medicine. The diagnostic antigen is capsular polysaccharide, which is shed from cryptococcal cells and can be easily detected in cerebrospinal fluid and serum by antibody-based assays such as bead agglutination and lateral flow assays. It is interesting that while antigen detection is an excellent test for aiding the diagnosis of disease, it is less useful for following the response to therapy because infection is associated with large polysaccharide deposits in cells and tissues; thus, the assay can remain positive even when antifungal therapy is successful. Hence, the diagnostic options for cryptococcosis are significantly better than those for other fungal diseases; however, prompt and proper use requires awareness and consideration of this disease by health care providers. There are new technologies in development that can enhance diagnostic tools such as rapid diagnosis and species differentiation based on CRISPR-Cas12a technology [270]. A promising area of investigation is the detection of fungal products in the brain using nuclear magnetic resonance imaging [271]. In this regard, MRI focusing on fungal trehalose has been used to discriminate between brain tumors and fungal cryptococcomas [272].

### Therapeutic considerations

Viewing the therapy of cryptococcosis from an ecological perspective involves the use of drugs to kill fungal cells or interfere with their replication in the ecologic niche defined by the human or animal host. Cryptococcosis was invariably fatal until the introduction of amphotericin B in the late 1950s. By the late 1960s, mortality with therapy had been reduced to 30%, but relapses were frequent, and these had a poor prognosis [273]. In the 1970s, the addition of 5-Fluorocytosine to amphotericin B resulted in additional incremental improvements in cryptococcosis therapy, but mortality remained at about 20% for those affected [274]. In the 1980s, a major problem was AIDS-related cryptococcosis, which was considered incurable and required maintenance therapy with antifungal agents to prevent relapse. The development of fluconazole as an effective oral antifungal agent in the late 1980s was a tremendous therapeutic advance because it could be used to suppress infection [275]. Currently, amphotericin B, 5-fluorocytosine, and fluconazole are the mainstays of cryptococcosis therapy. Fluconazole heteroresistance can emerge during therapy and is associated with aneuploidy [77,276]. Although other agents in the form of newer azoles have been shown to have activity against CN and amphotericin B preparations have been improved by incorporation into liposomes, the major therapeutic regimen for cryptococcosis has remained consistently dependent on amphotericin B for almost 70 years. Paradoxically, more progress has been made in the treatment of AIDS-related cryptococcosis than cryptococcosis in HIV-negative individuals. This reflects the differences in the biology of the disease in patients with HIV, as well as the availability of large numbers of cases of HIV-associated cryptococcosis for clinical trials, which has led to the testing of numerous regimens, and mortality in this population is now less than 10% when appropriately treated. In patients with HIV-associated cryptococcosis, the damage is primarily caused by the fungus, whereas in the non-HIV population, there is a significant immune component to the pathology that complicates treatment options and causes a relatively worse prognosis. It suffices to say that we have successful therapeutic regimens for cryptococcosis, but these remain suboptimal since the disease continues to have an unacceptably high mortality rate. A major cause of death due to cryptococcosis is progressive intracranial pressure with compression of the brain and neural structures. Although repeated lumbar punctures to relieve pressure have played an important role in the management of cryptococcosis [277], therapies to address the root causes of brain edema are lacking.

### Current and future challenges

Tremendous progress has been made in the past three decades, especially in the areas of molecular biology, genomics and cell biology. However, the therapy of cryptococcosis remains unsatisfactory with an unacceptable high mortality even when using state of the art antifungal regimens. Current and future challenges in the cryptococcal field include the need to prevent disease in high-risk individuals, improve therapy to further reduce mortality, and understand certain aspects of the biology and pathogenesis of this pathogenic fungus. For each of these challenges to be met, additional basic scientific and clinical studies are needed to better understand the pathogenesis of cryptococcosis and the biology of CN. As hopefully this essay has made clear, understanding the virulence and pathogenesis of cryptococcal species requires detailed knowledge of their ecology. Much of what we know about the ecology and the conditions that promote the growth of CN and CG in the environment was obtained decades ago, and there is an important need to revisit those studies using modern biological tools. In the realm of therapeutics, the greatest challenge for clinicians is to know where the patient is in the damage-response curve (Figure 3), which would allow the addition of immunotherapy to antifungal therapy. In this regard, individuals on the left horn of the parabola, such as those with HIV-related cryptococcosis, could benefit from adjunctive therapy to stimulate the immune system, as suggested by more favorable responses in those treated with IFN-gamma [229]. On the other hand, individuals in the right horn of the parabola, such as those with non-HIV-related cryptococcosis, could benefit from adjunctive therapy to downregulate the immune response, which has shown promise in some individuals with severe disease [278,279]. On the basic science front, the biology of CN poses some unique questions in cell biology that, when answered, can reveal new processes. For example, our understanding of how the capsule is assembled, removed, and maintained remains in its infancy, and when solved, is likely to provide new paradigms for eukaryotic capsular construction. Similarly, details about the cell wall architecture, the mechanism of cell wall transit by extracellular vesicles and the process of cell wall melanization remain unsolved problems in the field. John Perfect once referred to CN as the “sugar coated killer with designer genes” [280], a description that remains apt today and encompasses the major challenges confronting the cryptococcal field.

## Data Availability

This is a review paper. There is no primary data, and no data were generated during this work. All the information is in the text and references.
